# Supplementary material for: WASp modulates RPA function on single-stranded DNA in response to replication stress and DNA damage
Source: Nat Commun. 2022 Jun 29;13:3743. doi: 10.1038/s41467-022-31415-z (PMC9243104; doi:10.1038/s41467-022-31415-z)
Supplement: Supplementary file 1 — Supplementary Information [file 41467_2022_31415_MOESM1_ESM.pdf]

Western blot

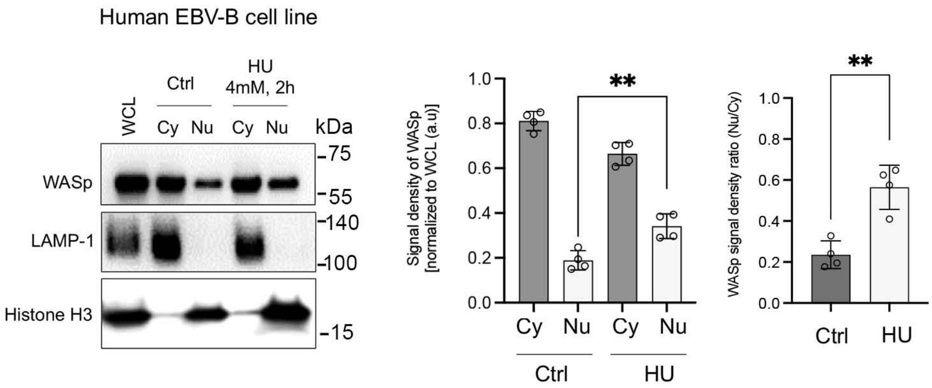

Fig. S1. WASp localization pattern in nuclear and cytoplasmic compartments. Western blots showing the relative abundance of WASp in the nuclear and cytoplasmic fractions of human B cells under HU-induced DNA stress or no stress conditions. The gel densitometry quantification of the corresponding bands normalized to the total protein signal in whole cell extract (WCE) is shown to the right. The purity of nuclear and cytoplasmic fractions was monitored by Histone H3 and LAMP-1 respectively. The data is representative of 3 independent assays. mean+SEM **$p<0.01$ by Mann-Whitney unpaired two-sided nonparametric. a.u. denotes arbitrary units. Source data are provided as a Source Data file.

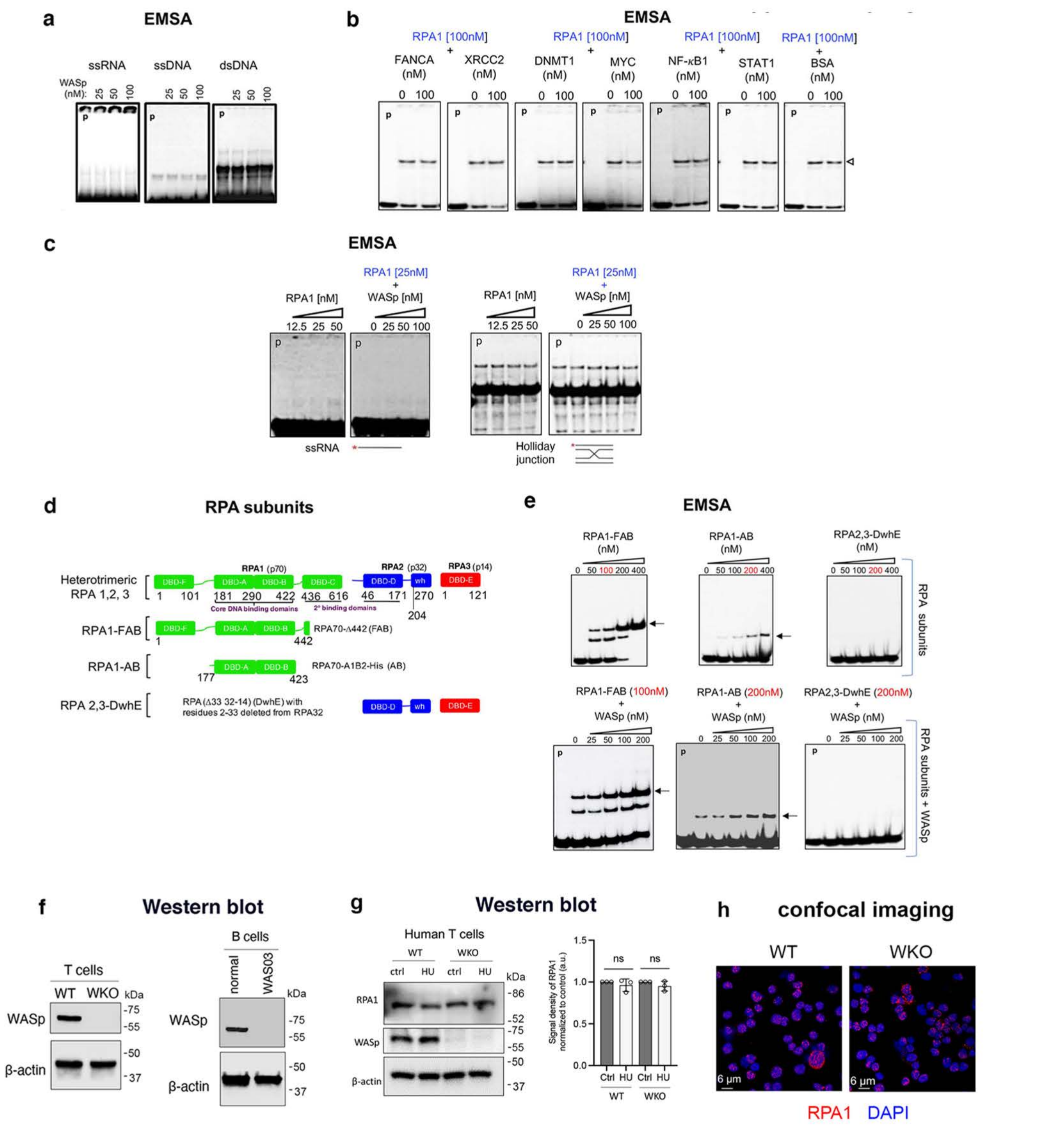

Fig. S2: **EMSA binding profiles of heterotrimeric RPA and RPA subunits.**

a, EMSA studies showing lack of WASp binding, at the indicated increasing concentrations (nM), to the indicated nucleic-acid conformations. ss, single-stranded; ds, double-stranded; p, probe only lane. The data is representative of n=2 independent assays.

b, EMSA studies showing lack of modulation of RPA1 binding to ssDNA by the indicated proteins.

c, EMSA studies showing lack of RPA1 binding to ssRNA or DNA Holliday junction, which are not ectopically upregulated by adding WASp even at high concentration (100nM). The data is representative of n=2 independent assays.

d, shown are 3 subunits of heterotrimeric RPA molecule (RPA1, RPA2, RPA3) and the indicated truncated mutants. The data is representative of n=2 independent assays.

e, EMSA studies showing ssDNA binding activities of the indicated RPA subdomain mutants, and the effect of adding WASp to these mutants. The data is representative of n=2 independent assays.

f, Western blots reporting of the expression of WASp in total cell lysates of the indicated cell types. WT, wild type and WKO, *WAS* knock-out T cell lines are isogenic pair; B cells, normal donor and WAS03 patient derived, EBV-B cell lines. The data is representative of n=3 independent assays.

g, Western blot showing the expression of the indicated proteins in the whole cell extracts of human T cells, WT or WKO, either treated with HU or untreated control. The corresponding gel densitometry data is shown to the right. The data is representative of n=3 assays, mean +SEM ns= nonsignificant p value by Mann-Whitney unpaired two-sided nonparametric. a.u. denotes arbitrary units. Scale bar: 6μm. Source data are provided as a Source Data file.

h, Confocal IF images of T cells (WT and WKO), showing RPA1 nuclear localization in the invitro culture condition. Scale bar: 6μm.

a **Chromatogram of GFP-tagged vectors containing Wt-WASp or WASp mutant deleted of RPA1-binding motif**

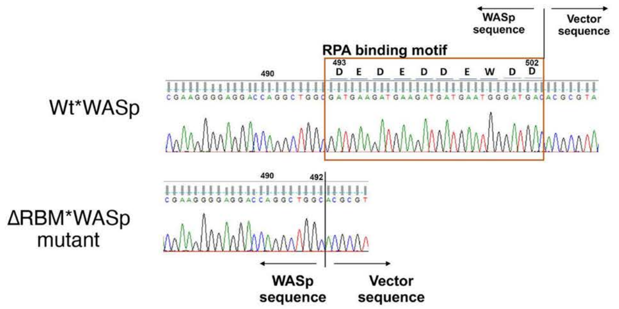

b **Flow cytometry**

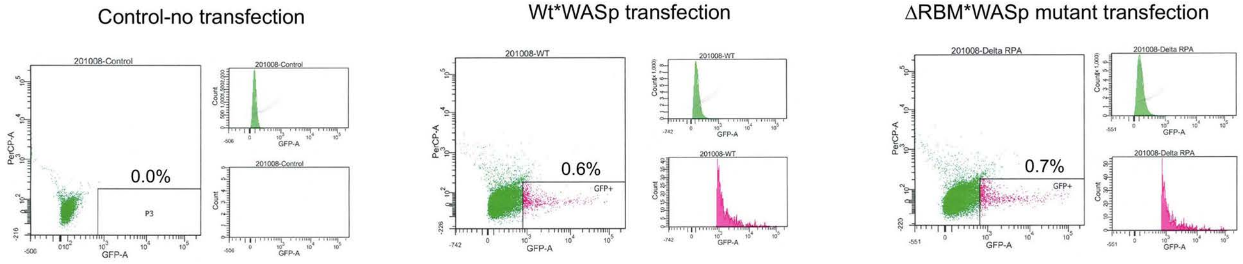

Fig. S3: **Verification of RPA1-binding motif (RBM1) deleted mutant of WASp. a,** DNA sequence chromatogram of the mutant showing the location of the RBM1 (bounded by red box) and the boundary separating the vector from WASp sequences (by arrows). **b,** Dot plot and histograms showing the gating parameters applied to sort by FACS, GFP +ve cells transfected with either GFP-tagged WT*WASp or the ΔRBM1*WASp mutant.

DNA fiber assay

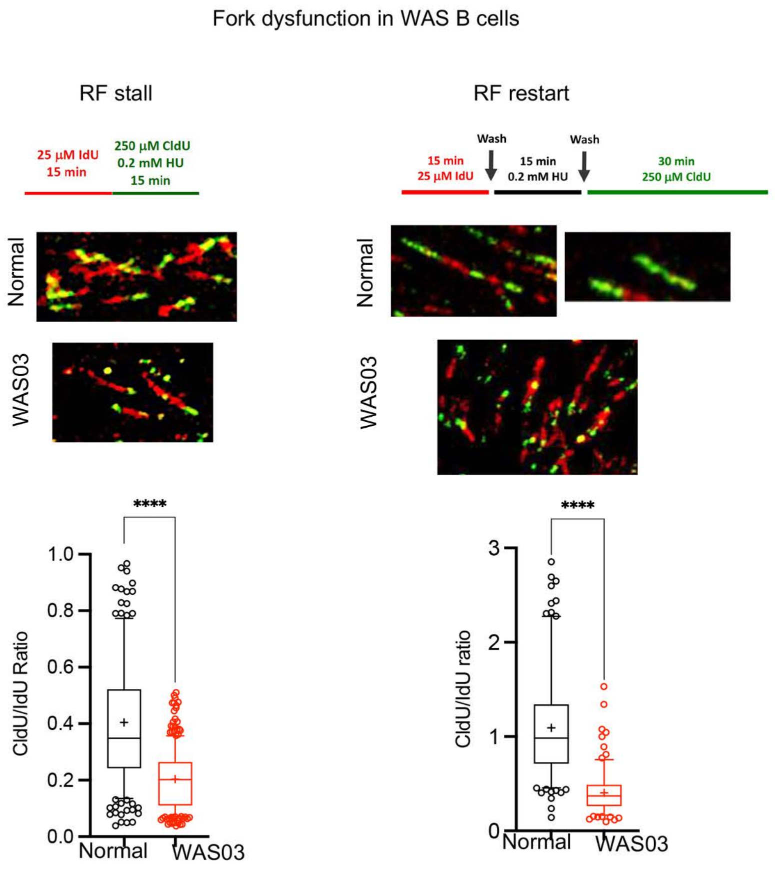

Fig. S4. DNA fiber assay. Fork dysfunction in WAS patient B cells. DNA fiber assays showing 2 different labeling protocols, their representative replication track images, and their corresponding RF statistics in human B cells (WT, WAS03 patient-derived) under unperturbed (left panel) or HU-perturbed (right panel) conditions. The box-and-whisker plots (whiskers @10-90%, horizontal bar denotes median, "+" denotes mean) are from n=150 tracks analyzed from 3 independent experiments. Mann-Whitney unpaired two-sided nonparametric p-value: ****<0.0001; ns, nonsignificant.

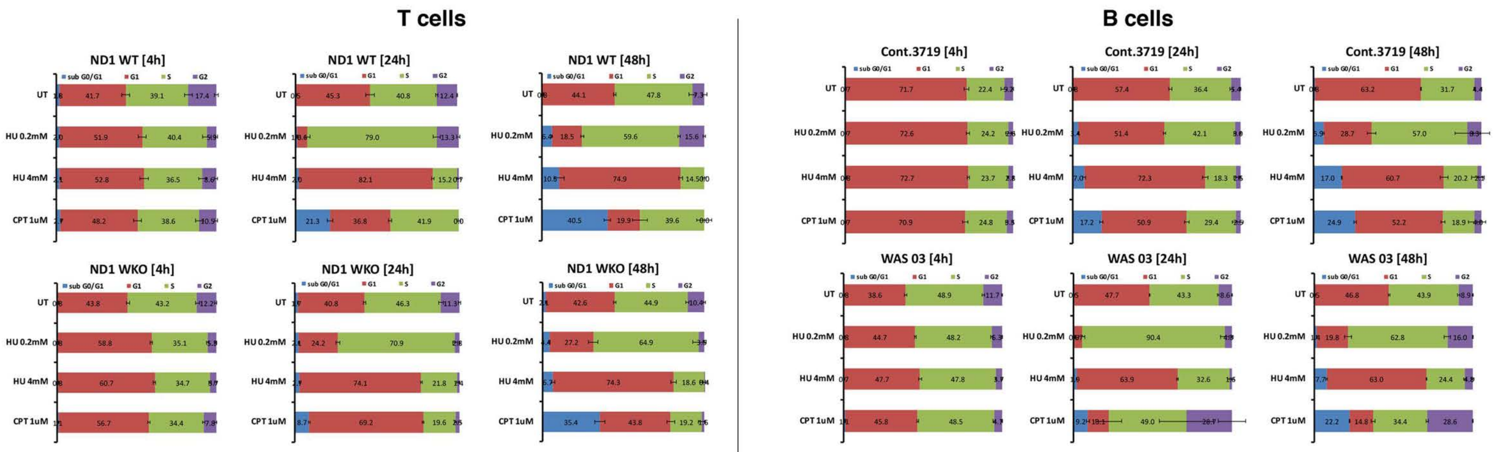

low dose HU (replication arrest)
normal T cells: S arrest (24hr, 48hr)
WAS T cells: S arrest (24hr, 48hr)

High dose HU (DSB+ RS)
normal T cells: G1 arrest (24hr, 48hr): i.e., delay entry to S-phase
WAS T cells: G1 arrest (24hr, 48hr)

CPT
normal T cells: G1, S arrest (24 hr)
WAS T cells: G1 arrest (24 hr)

low dose HU (replication arrest)
normal B cells: S arrest (48hr)
WAS B cells: S arrest (24hr, 48hr) early capture of profound S arrest

High dose HU (DSB+ RS)
normal B cells: G1 arrest (24hr, 48hr)
WAS B cells: G1 arrest (24hr, 48hr)

CPT
normal B cells: G1 arrest (24-48hr)
WAS B cells: S, G2/M arrest (24-48hr), G2 checkpoint activation

Fig. S5: Cell cycle analyses. Cell cycle profiles of T and B cells (WT or WASp-deficient) treated with the indicated genotoxins and analyzed at the 3 indicated time-points after genotoxin damage induction or untreated control (UT). The data shows mean+SD from n=3 independent experiments. Key summary describing the bar graph data are provided under the figure.

Supplementary Fig. S6

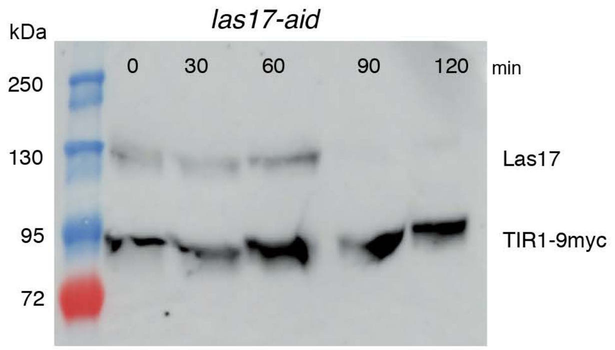

Fig. S6. Las17 depletion in *las17-aid* degron strain. Western blot using the anti-Myc antibody of the *las17-aid* strain at different times after 1 mM auxin addition. The strain was constructed to express the auxin-inducible degron and myc epitopes fused to las17 for its detection. The data is representative of 2 independent assays.

Table S1: KEY RESOURCES TABLE

REAGENT or RESOURCE	SOURCE	IDENTIFIER
Antibodies		
RPA1	Origene	TA308716
RPA2	Origene	TA500786
WASP	proteintech	10987-I-AP
WASp (F8)	Santa Cruz	sc-365859
WASp (D1)	Santa Cruz	sc-5300
Phospho RPA32 (S33)	Bethyl	A300-246A
Chk1	Santa Cruz	sc-8408
Phospho-Chk1(Ser345)	CST	2348S
p-Histone H2A.X(Ser139)	Santa Cruz	sc-101696
p-Histone H2A.X(Ser139)	Santa Cruz	sc-517348
ATR	Santa Cruz	sc-515173
ETAA-1	Invitrogene	PA5-103838
Actin	proteintech	66009-1
IgG	Santa Cruz	sc-66931
Goat anti-rabbit IgG peroxidase conjugate	Sigma	A 6154
Goat anti-mouse IgG (H+L), HRP conjugate	proteintech	SA00001-1
Goat anti-rabbit IgG (H+L), HRP conjugate	proteintech	SA00001-2
Rabbit Anti-Biotin	Bethyl	A150-109A
Mouse Anti Biotin	Jackson	200-00-211
Duolink® In Situ PLA® Probe Anti-Rabbit PLUS, Affinity purified Donkey anti-Rabbit IgG (H+L)	MilliporeSigma	DUO92002
Duolink® In Situ PLA® Probe Anti-Mouse MINUS, Affinity purified Donkey anti-Mouse IgG (H+L)	MilliporeSigma	DUO92004
Rat anti-BrdU	Abcam	Ab6326
Mouse anti-BrdU	BD Biosciences	347580
Anti-mouse IgG1 Alexa Fluor 547	Invitrogen	A21123
Anti-rat Alexa Fluor 488	Invitrogen	A21470
Mouse monoclonal c-Myc	TaKara	631202
Recombinant proteins		
RPA1	Origene	TP302066
RPA2	Origene	TP305715
MAX	Origene	TP320343
Arp3	Origene	TP308460
Arp2	Abcam	ab217837
Ribonuclease A	Sigma-Aldrich	R6513
Dynabeads protein A	Thermo Fisher	10001D
RNase H	NEB	M0297L
Proteinase K	Roche	31115852001
Ambion RNase III	Thermo Fisher	AM2290
Reagents		
0.8 mg/ml of O-phenylenediamine	Sigma	P5412-50TAB

0.05 M phosphate-citrate buffer containing 0.03% sodium perborate	Sigma	P4922-100CAP
Goat anti-rabbit IgG peroxidase conjugate	Sigma	A 6154
30% Acylamide/Bis solution (37.5:1)	Bio-Rad	161-0158
γ-[^{32}P]-ATP	Perkin-Elmer	BLU502A250UC
Nick column sephadex G-50 DNA grade	GE Healthcare	17-0855-02
T4-polynucleotide kinase	Promega	M4108
5 x TBE Buffer	Sigma	T6400-4L
Precision red advanced protein assay reagent	Cytoskeleton	DV02-A
Nucleofactor Kit V	Lonza	VCA-1003
Nucleofactor Kit L	Lonza	VCA-1005
Duolink® In Situ Detection Reagents Orange	Sigma	DUO92007
Biotin-dPEG7-azide	Quanta Biodesign	10825-100MG
EdU	Cayman Chem	20518
CldU	Cayman Chem	18155
IdU	Cayman Chem	20222
RIPA buffer	Sigma	R0278
Benzonase	Millipore	706643
VECTASHIELD® Antifade Mounting Medium with DAPI	Vector	H-1200-10
Hydroxyurea	Sigma	H8627
Camptothecin	Cayman Chem	11694
Methyl methanesulfonate (MMS)	Fluka	G4294
CometAssay Reagent Kit	Trevigen	4250-050-K
Trans-Blot Turbo RTA Mini 0.2 µm PVDF Transfer Kit	Bio-Rad	1704272
Halt™ Protease and Phosphatase Inhibitor Cocktail (100X)	Themo-Fisher	78440
2x Laemmli Sample Buffer	Bio-Rad	1610737
Streptavidin agarose	Millipore	69203
Zymoliase 20T	USB Biological	Z1000
Dynabeads protein A	Thermo Fisher	10001D
iTAq Universal SYBR-green supermix	Biorad	1725125
Phusion High-Fidelity DNA polymerase	Finnzymes	F-530L
BsrGI	NEB	R0575S
EcoRI	NEB	R0101S
XbaI	NEB	R0145S
HindIII	NEB	R0104S
SspI	NEB	R0132S
Critical commercial assays		
NucleoSpin Gel and PCR Clean-up	Macherey-Nagel	740609
Experimental models: Cell lines		
ND1	This paper	N/A
ND1 WKO	This paper	N/A
Cont. 3719	Coriell Institute	ND03719
WAS 03	Coriell Institute	ID00003
Experimental models: Organisms/strains		

Yeast Strain	This paper	See Table S4
Recombinant DNA		
WASP-CRISPR/CAS9-GFP plasmid	Santa Cruz biotech	sc-400712-KO-2
Oligonucleotides		
Primer: for genomic *WAS* knockout: Forward: 5-GGAATCAGAGGCAAAGTGGA-3	This paper	N/A
Primer: for genomic *WAS* knockout: Reverse: 5-TAAAATGGGTACCCCACTG-3	This paper	N/A
Primer: for mRNA *WAS* knockout: Forward: 5-CCCTCGTGCAGGAGAAGATA-3	This paper	N/A
Primer: for mRNA *WAS* (knockout): Reverse: 5-CAGAACGACCCTTGTTACCC-3	This paper	N/A
Primer: for DRIP qPCR: *PDR5* Forward: 5-GTCAGAGGCTATATTTCACTGGAGA-3	This paper	N/A
Primer: for DRIP qPCR: *PDR5* Reverse:	This paper	N/A
5- TACGTCTTGTTTCGGCCTTAATC-3	This paper	N/A
Primer: for DRIP qPCR: *SPF1* Forward:	This paper	N/A
5- CCCGTGGTAAACCTTTAGAAA-3	This paper	N/A
Primer: for DRIP qPCR: *SPF1* Reverse: 5-ATATGAACGGCAAATTGAGAC-3	This paper	N/A
Primer: for Las17-IAA degron: Forward: 5GAAAAACTAAAGTGGGAGCTCATGACGATATGG ACAATGGTGATGATTGGCGTACGCTGCAGGTCGA C-3	This paper	N/A
Primer: for Las17-IAA degron: Reverse: 5- TTACATATTTTCTATAACAGTAGTTTCATCTTTGTT TGCATTCCAATCGATGAATTCGAGCTCG-3	This paper	N/A
Software and algorithms		
ImageJ		
Other		

Table S2. Sequences of the oligonucleotides used for DNA substrate.

Name	Sequence	Identifier
XO1	GACGCTGCCGAATTCTACCAGTGCCTTGCTAGG ACATCTTTGCCCACCTGCAGGTTCACCC	Gari.K et al 2008
XO4	ATCGATAGTCGGATCCTCTAGACAGCTCCATGTA GCAAGGCACTGGTAGAATTCGGCAGCGT	Gari.K et al 2008
D1	GGGTGAACCTGCAGGTGGGCAAAGATGTCCTAG CAAGGCACTGGTAGAATTCGGCAGCTGC	Gari.K et al 2008
DS3	CATGGAGCTGTCTAGAGGATCCGACTATCGA	Gari.K et al 2008
DS4	TAGCAAGGCACTGGTAGAATTCGGCAGCGT	Gari.K et al 2008

These oligonucleotides were adopted from a design by Gari et al (2008).

Table S3. Combinations of oligonucleotides annealed to create the DNA substrates

DNA substrates	Annealed oligonucleotides	Identifier
Single-stranded DNA (ssDNA)	XO1	Gari.K et al 2008
Double-stranded DNA (dsDNA)	XO1 + D1	Gari.K et al 2008
3'-tailed DNA	XO1 + DS4	Gari.K et al 2008
Splayed arm DNA	XO1 + XO4	Gari.K et al 2008
3'-flap DNA	XO1 + XO4 + DS3	Gari.K et al 2008

These Combinations of oligonucleotides were adopted from a design by Gari et al (2008).

Table S4. Yeast strains used in this study.

Strain	Genotype	Source
BY4741	*MAT***a** *his3Δ1 leu2Δ0 met15Δ ura3Δ0*	Euroscarf
BY-LAS17-14	BY4741 *las17-14*	Li et al, 2011
BY-ARP3-D11A	BY4741 *arp3-D11A*	Li et al, 2011
WRFA17-2C	*MATα RFA1-8ala-YFP RAD5*	This study
WRFA17-1C	*MATα RFA1-8ala-YFP RAD5 las17-14::Kan*	This study
YMK612	*Mat***a** *leu2-3,112 trp1-1 can1-100 ura3-1 ade2-1 his3-11,15 ura3-1::ADH1-AtTIR1-9myc*	Nishimura et al, 2009
YMK-L17-D	YMK612 *Matα ura3-1::ADH1-AtTIR1-9myc las17-aid*	This study
