# Supplementary figures and images for: WASp modulates RPA function on single-stranded DNA in response to replication stress and DNA damage
Source: Nat Commun. 2022 Jun 29;13:3743. doi: 10.1038/s41467-022-31415-z (PMC9243104; doi:10.1038/s41467-022-31415-z)

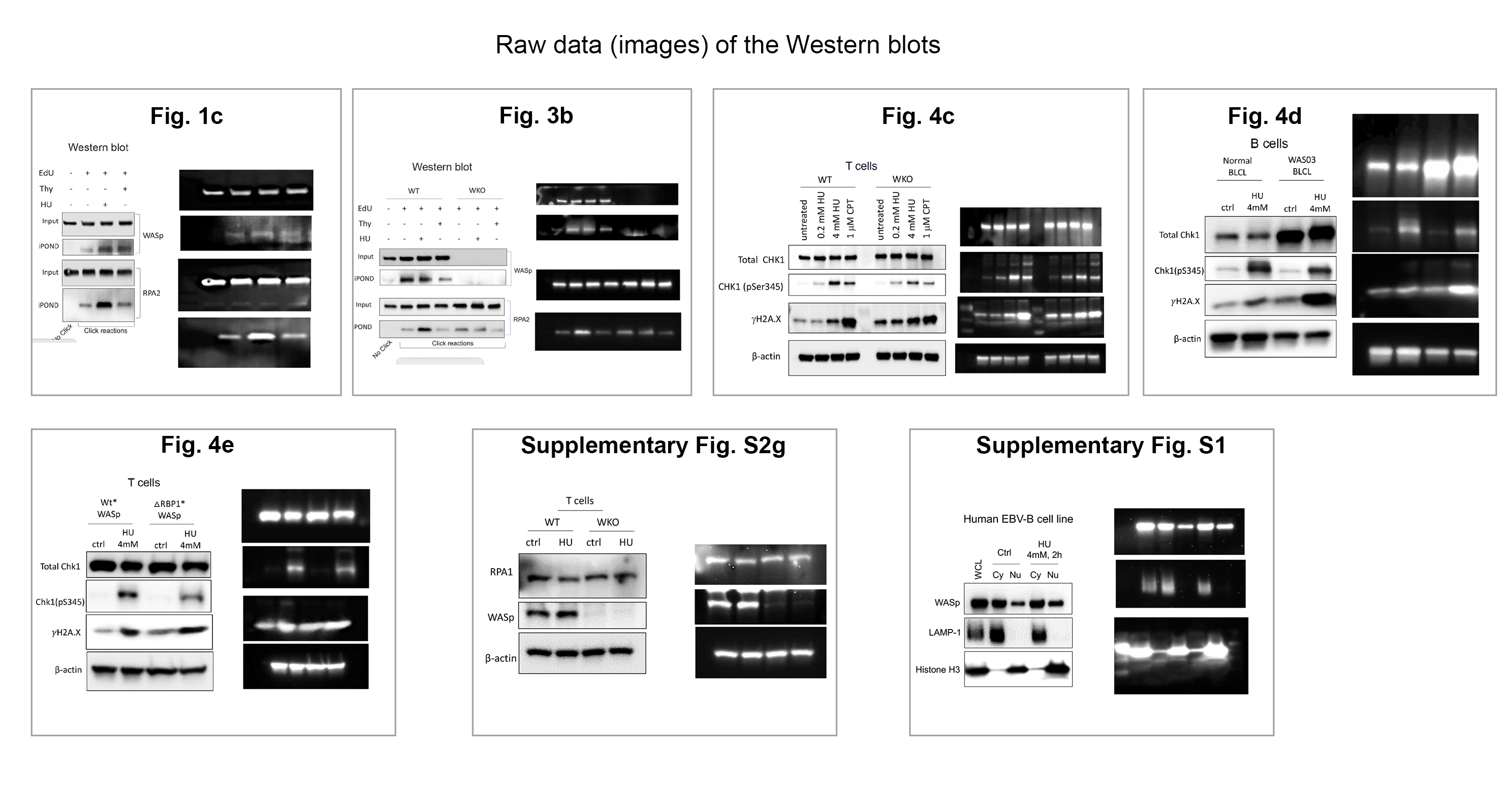

Supplement: Supplementary file 4 — Source Data [file 41467_2022_31415_MOESM4_ESM.zip › Source data file.tif]
